# Case Report: Application of multimodal imaging in the diagnosis and treatment evaluation of primary cardiac lymphoma

**DOI:** 10.3389/fcvm.2026.1732686

**Published:** 2026-03-05

**Authors:** Hanxing Shi, Jing Liang

**Affiliations:** 1 Department of Radiology, Nanjing Drum Tower Hospital Clinical College of Nanjing University of Chinese Medicine, Nanjing, China; 2 Department of Radiology, Nanjing Drum Tower Hospital, Affiliated Hospital of Medical School, Nanjing University, Nanjing, China

**Keywords:** cardiac magnetic resonance imaging, computed tomography, echocardiography, positron emission tomography-computed tomography, primary cardiac lymphoma

## Abstract

Primary cardiac lymphoma (PCL) is an extremely rare malignancy. This report describes a 76-year-old woman who presented with chest tightness, exertional dyspnea, and nocturnal orthopnea. Multimodal imaging (echocardiography, CT, CMR, PET) was central to the diagnostic process, enabling precise tumor localization, non-invasive tissue characterization, and disease staging, thereby raising strong suspicion for PCL. The diagnosis of primary cardiac diffuse large B-cell lymphoma (non-germinal center subtype) was subsequently confirmed by histopathology. The patient achieved complete remission after five cycles of chemotherapy, which was confirmed on 6-month follow-up imaging, with no recurrence over 65 months of surveillance. This case highlights that multimodal imaging provides complementary information crucial for the comprehensive evaluation of PCL, serving as a cornerstone for evidence-based clinical decision-making throughout the diagnostic and therapeutic continuum, from initial suspicion and staging to treatment response assessment.

## Introduction

Primary cardiac lymphoma (PCL) is a relatively rare cardiac tumor, accounting for approximately 1.3% of all primary cardiac tumors and about 0.5% of extranodal lymphomas (1–3). In 2015, the World Health Organization (WHO) provided a clear definition of PCL: narrowly defined as “an extranodal lymphoma involving only the heart or pericardium,” and broadly defined as “a large lymphoma mass in the heart, with or without secondary small lesions in other areas of the body (4).” The most common pathological type is diffuse large B-cell lymphoma (DLBCL), which predominantly affects the right heart chambers (especially the right atrium) (1, 2). Patients with PCL often present with non-specific symptoms such as dyspnea, chest pain, syncope, and arrhythmias (5). If complications such as pulmonary embolism occur, the disease progression can be rapid, leading to a poor prognosis (5). Therefore, early identification, accurate diagnosis, and timely intervention are crucial for improving patient outcomes. In modern clinical practice, the integrated application of multimodal imaging techniques has become a core approach for evaluating and diagnosing such complex cardiac mass lesions. This report will systematically elaborate on the synergistic value and clinical significance of multimodal imaging in the diagnosis, treatment decision-making, and long-term follow-up of PCL, using a pathologically confirmed case as an example.

## History of presentation

A 76-year-old female presented with a two-week history of progressive symptoms including chest tightness after drinking water and exertional dyspnea (relieved by rest), which eventually progressed to orthopnea accompanied by cough with white frothy sputum. Despite undergoing ultrasound-guided thoracentesis for pleural effusion drainage, the patient's retrosternal tightness persisted unrelieved.

## Investigations

Initial laboratory tests revealed the following abnormalities: elevated neutrophil percentage at 81.7% (reference range 40%–75%), increased neutrophil count of 7.2 × 10^9^/L (reference range 1.8–6.3 × 10^9^/L), monocytosis with a count of 0.7 × 10^9^/L (reference range 0.1–0.6 × 10^9^/L), elevated B-type natriuretic peptide level of 244.0 pg/mL (reference range 5–100 pg/mL), and increased D-dimer concentration of 4.31 mg/L (reference range < 0.5 mg/L).

**Figure 1:**
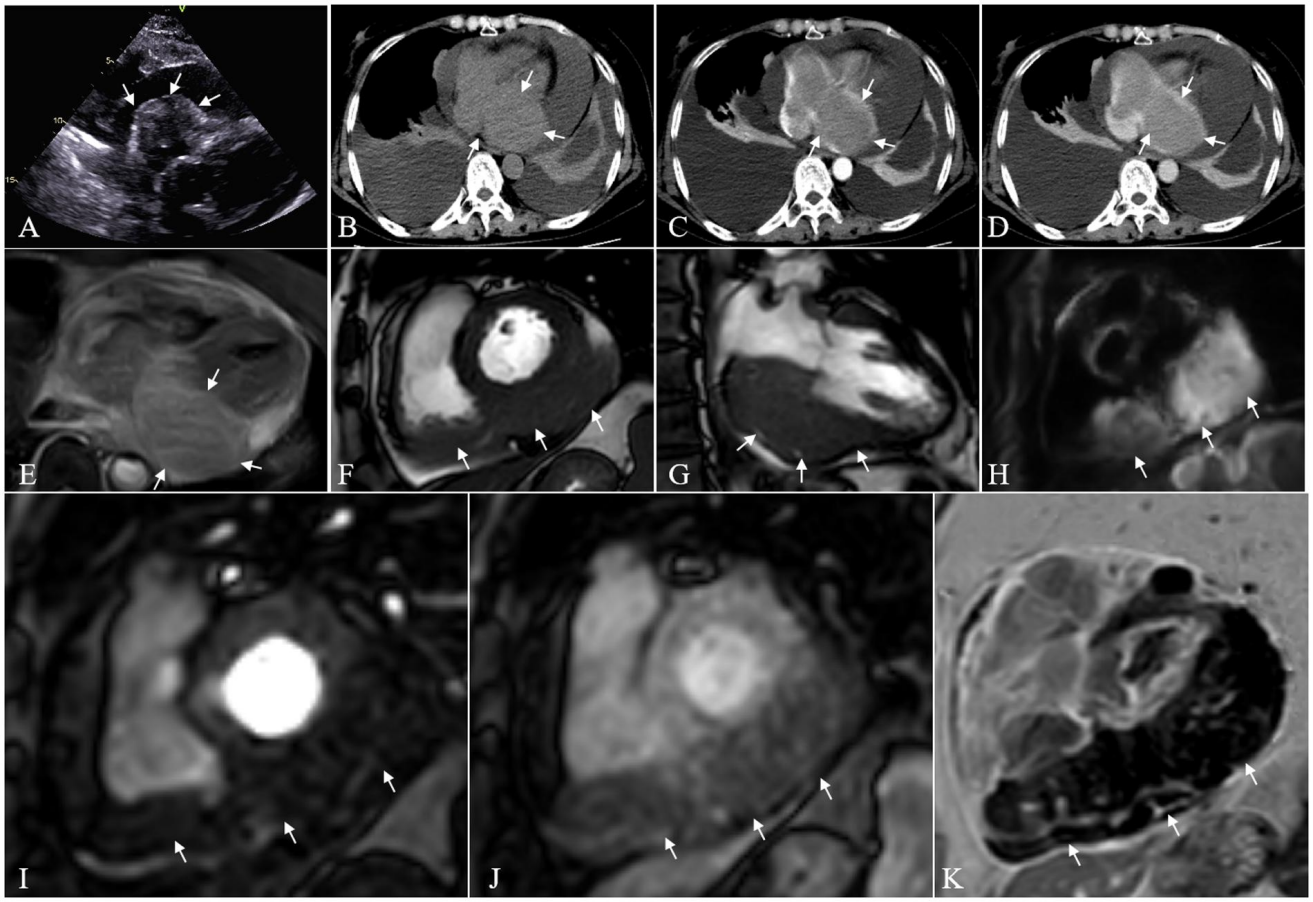
**(A)** Echocardiography reveals an irregular, heterogeneous iso-hypoechoic mass within the right atrium, demonstrating ill-defined margins with the lateral atrial wall and tricuspid annulus. **(B)** Non-contrast chest CT identifies a homogeneous soft tissue mass at the atrioventricular junction (CT value: 41 HU). **(C)** Arterial-phase contrast-enhanced CT exhibits moderate homogeneous enhancement of the lesion (CT value: 76 HU). **(D)** Venous-phase shows reduced enhancement compared to adjacent myocardium (CT value: 80 HU). **(E–G)** Cardiac magnetic resonance (CMR) demonstrates extensive soft tissue masses spanning bilateral atrioventricular junctions. **(E)** T2-weighted imaging displays mildly hyperintense signal relative to normal myocardium. **(F)** The mass exhibits inward protrusion into the atrial cavity and outward pericardial infiltration. **(H)** Diffusion-weighted imaging reveals significant diffusion restriction. **(I,J)** First-pass perfusion imaging demonstrates progressive enhancement of the lesion with lower intensity than adjacent normal myocardium. **(K)** Delayed enhancement (10-minute post-contrast) displays heterogeneous enhancement pattern with scattered linear hyperenhancement within the mass.

Electrocardiography demonstrated frequent atrial premature beats. Echocardiography showed right atrial enlargement with an irregular, hypoechoic immobile mass demonstrating poorly defined borders with the right atrial lateral wall and tricuspid annulus (Figure 1A).

Chest CT was performed without electrocardiographic gating. A non-ionic iodinated contrast agent was administered as a bolus via an antecubital vein at a flow rate of 4.0 mL/s; the dose was weight-based (1.0 mL/kg). Chest non-contrast CT revealed an isodense soft tissue mass at the atrioventricular junction with uniform attenuation (approximately 47 Hounsfield units), showing similar density and ill-defined borders with the myocardium (Figure 1B). Contrast-enhanced CT demonstrated homogeneous moderate enhancement of the mass, with attenuation values of 75 HU in the arterial phase and 78 HU in the venous phase, which were lower than those of the adjacent myocardium (Figures 1C,D). The mass extended to the orifice of the inferior vena cava and enveloped the right coronary artery. Additional findings included mediastinal lymphadenopathy, moderate pericardial effusion, and bilateral massive pleural effusions.

Cardiac magnetic resonance (CMR) demonstrated an extensive soft tissue mass at the atrioventricular junction, which appeared slightly hyperintense on fat-suppressed T2-weighted imaging (T2WI) compared to normal myocardium (Figure 1E). The mass protruded into the cardiac chamber while simultaneously exhibiting outward invasion of the pericardium (Figures 1F,G). Diffusion-weighted imaging (DWI) revealed marked diffusion restriction (Figure 1H). First-pass perfusion imaging showed progressive enhancement of the lesion with lower intensity than adjacent normal myocardium (Figures 1I,J). Delayed enhancement imaging (10 min post-gadolinium administration) demonstrated heterogeneous enhancement pattern with scattered linear hyperenhancement within the mass (Figure 1K).

**Figure 2:**
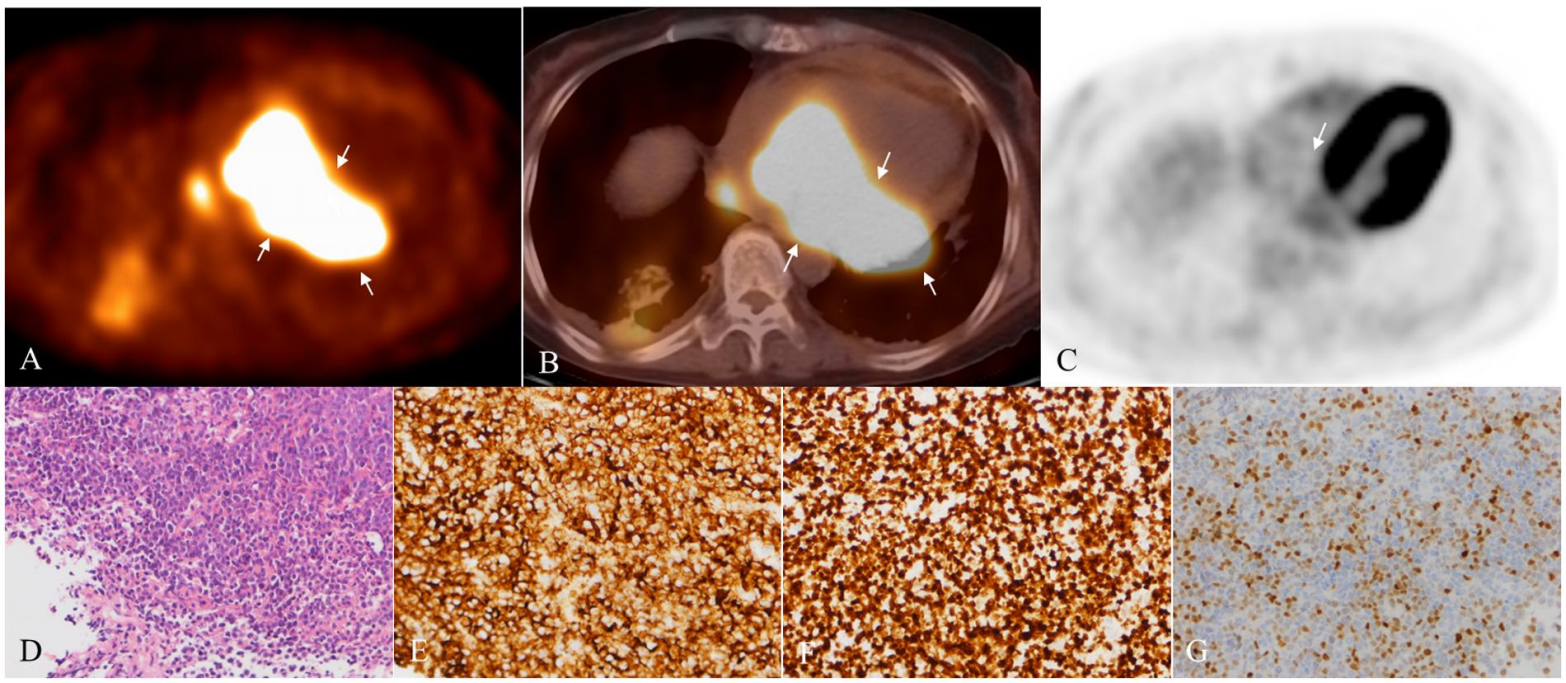
**(A,B)** Pre-treatment 18F-FDG PET/CT identifies a hypermetabolic soft tissue mass at the atrioventricular junction (SUVmax: 20.3). **(C)** Post-treatment FDG-PET/CT (5 cycles of R-miniCHOP) confirms complete metabolic resolution. **(D–F)** Histopathological analysis confirms primary cardiac diffuse large B-cell lymphoma. **(D)** Hematoxylin and eosin (H&E) staining shows sheets of atypical lymphoid cells. **(E)** Immunohistochemistry reveals strong membranous CD20 expression. **(F)** Ki-67 labeling index highlights high proliferative activity (90%). **(G)** Nuclear MUM1 expression supports non-germinal center B-cell subtype.

Positron Emission Tomography-Computed Tomography (PET-CT) imaging demonstrated a hypermetabolic soft tissue mass (SUVmax 20.3) localized at the atrioventricular junction (Figures 2A,B), accompanied by multiple 18F-fluorodeoxyglucose (18F-FDG) -avid mediastinal lymph nodes.

Based on the aforementioned multimodal imaging findings, the evidence supporting the diagnosis of primary cardiac lymphoma is both complete and characteristic. First, CMR demonstrated a mass with a typical “bidirectional infiltration” pattern, protruding into the cardiac chamber while simultaneously invading outward into the pericardium, consistent with the diffuse growth behavior of lymphoma. Second, DWI showed marked diffusion restriction, suggesting high tumor cellularity, which corresponds to the histological features of lymphoma. Third, fat-suppressed T2WI displayed homogeneous slightly hyperintensity, and contrast-enhanced sequences indicated absence of necrosis or hemorrhage. Fourth, PET-CT revealed significantly increased metabolic activity within the lesion (SUVmax 20.3), aligning with the hypermetabolic nature of lymphoma, features in keeping with the imaging profile of lymphoma. In summary, the combination of infiltrative growth, homogeneous signal intensity, diffusion restriction, characteristic enhancement patterns and hypermetabolism, collectively constitutes the imaging evidence for diagnosing primary cardiac lymphoma.

## Management

Comprehensive preoperative multimodal imaging analysis raised suspicion of PCL with invasion into adjacent cardiac structures. Given unresectability, ultrasound-guided biopsy targeting the right atrial mass near the inferior vena cava orifice yielded two core samples (needle position verified by real-time sonography). Histopathological examination confirmed the diagnosis of diffuse large B-cell lymphoma (DLBCL, non-germinal center B-cell subtype) with characteristic immunohistochemical features: a high proliferation index (Ki-67: 90%), strong CD20 positivity, and MUM1 positivity (Figures 2D–G). Given the patient's advanced age, an individualized R-miniCHOP regimen (rituximab plus reduced-dose cyclophosphamide, liposomal doxorubicin, vindesine sulfate, and prednisone) was administered after diagnosis. The specific dosages were adjusted according to the patient's performance status and organ function. The actual medications in the first cycle included rituximab 600 mg, cyclophosphamide 520 mg, liposomal doxorubicin 20 mg, and vindesine sulfate 2 mg; prednisone acetate tablets were given orally at 50 mg/day on days 1–5. During treatment, supportive care was provided concurrently, including cardioprotection, routine antiemetics, hydration with urinary alkalinization, and granulocyte colony-stimulating factor. The regimen was dynamically adjusted in subsequent cycles based on the patient's tolerance. The patient completed a total of 5 cycles of chemotherapy with good overall tolerance and no serious treatment-related adverse events.

## Follow-up

Post-chemotherapy PET/CT assessment confirmed complete metabolic response, characterized by total resolution of initial hypermetabolic lesions (Figure 2C) and absence of new FDG-avid foci. Throughout the 65-month follow-up, the patient maintained continuous progression-free survival (PFS), with serial imaging and clinical evaluations consistently demonstrating durable remission without evidence of disease recurrence.

## Discussion

PCL, a relatively rare malignancy (3), poses significant diagnostic and therapeutic challenges, as exemplified in this representative case. Multimodal imaging plays a pivotal role in PCL management (1, 6), with echocardiography serving as the first-line screening tool due to its noninvasive nature, accessibility, and real-time assessment capabilities for evaluating tumor location, morphology, mobility, and hemodynamic effects, including detection of secondary signs like pericardial effusion (7). However, inherent limitations, such as restricted spatial resolution, operator dependence, and acoustic window constraints, notably impair accurate assessment of tumor infiltration margins, benign/malignant differentiation, and extra-cardiac extension. In the present case, while echocardiography successfully identified the cardiac mass, it failed to precisely delineate tumor boundaries or adjacent tissue invasion, underscoring its inadequacy as a precise diagnostic modality, particularly for evaluating mediastinal and extracardiac structures where it exhibits substantial anatomical blind spots.

Recent advances in CT and CMR imaging have significantly enhanced the diagnostic evaluation of PCL. As highlighted by Asadian et al. (8), these sophisticated imaging modalities have become indispensable for PCL characterization through their multiparametric protocols and superior soft tissue contrast resolution. CT excels in delineating anatomical relationships with coronary and great vessels through its high spatial resolution and multiplanar reconstruction capabilities, while also assessing mediastinal lymphadenopathy and pericardial involvement, advantages well demonstrated in our case for determining tumor extent and detecting metastases. However, CT's diagnostic specificity is limited by beam-hardening artifacts in the right atrium (a common PCL location) and inherent soft tissue contrast limitations (9). On the contrary, CMR has become the gold standard (10) for evaluating cardiac masses due to its radiation-free nature, multi-parametric capabilities, and superior soft tissue contrast. In this case, CMR provided critical information for characterizing the lesion through systematic multi-sequence scanning: cine imaging revealed a mass located at the atrioventricular junction, demonstrating infiltrative growth accompanied by regional myocardial contractile dysfunction; fat-suppressed T2WI showed the mass as mildly hyperintense relative to normal myocardium. According to the summary by Motwani et al. (11), PCL often appears isointense to mildly hyperintense on T2WI, which is of significant differential diagnostic value compared to the markedly hyperintense signal of myxomas or the heterogeneous high signal of sarcomas with necrosis; diffusion-weighted imaging showed pronounced restricted diffusion of the tumor, indicating pathological features of high cellular density and reduced extracellular space, consistent with the typical presentation of lymphoma; first-pass perfusion imaging revealed progressive enhancement of the lesion, though the degree of enhancement was lower than that of the adjacent normal myocardium, this relative hypoperfusion pattern may be related to the tumor's dense cellular structure and relatively low microvascular density; delayed enhancement imaging demonstrated heterogeneous internal enhancement with scattered linear hyperenhancement, consistent with previous literature descriptions (1, 10, 11) and distinct from the pronounced enhancement seen in hypervascular tumors such as angiosarcoma or the lack of enhancement in thrombi. Integrating these imaging features, including infiltrative growth pattern, mild T2 hyperintensity, marked restricted diffusion, relative hypoperfusion, and mild heterogeneous delayed enhancement, the imaging findings are highly consistent with the typical pattern of primary cardiac lymphoma. This allows for effective differentiation from other common cardiac masses such as thrombi, myxomas, and angiosarcomas, thereby providing essential guidance for clinical diagnosis and therapeutic decision-making.

In clinical oncology, precise tumor staging serves dual critical purposes: it provides essential prognostic information while directly guiding stage-specific therapeutic strategies. 18F-FDG PET/CT has emerged as an indispensable tool for lymphoma management, offering unique advantages in initial staging, treatment response evaluation, and recurrence detection (12). Notably, PCL demonstrates characteristic PET/CT findings including elevated SUV and expanded metabolic tumor volumes, which enable more accurate biopsy targeting by identifying the most metabolically active tumor regions. Another critical application of 18F-FDG PET/CT in PCL management lies in treatment response evaluation, which constitutes an essential component of restaging for most lymphoma subtypes (13). In the current case, PET/CT imaging revealed significantly enhanced metabolic activity in the left atrial lesion, providing strong evidence supporting the malignant diagnosis while simultaneously ruling out distant metastasis. The complete metabolic resolution observed on follow-up PET/CT scans after chemotherapy confirmed therapeutic efficacy. However, the technique's limitations in spatial resolution result in certain constraints when evaluating the depth of myocardial infiltration. The metabolic-volume parameters provided by PET/CT must therefore be interpreted in conjunction with anatomical imaging findings (such as CMR or contrast-enhanced CT) for comprehensive clinical decision-making.

In comprehensive evaluation, various imaging modalities exhibit distinct advantages for assessing PCL: echocardiography serves as an ideal initial screening tool; CT provides superior anatomical localization and precise evaluation of tumor relationships with coronary arteries and major vessels; CMR excels in qualitative diagnosis and infiltration analysis; while PET/CT specializes in metabolic assessment and clinical staging. The combined application of multimodal imaging enables comprehensive characterization of tumor features and effectively overcomes diagnostic limitations inherent to single modalities. Of critical importance, current evidence demonstrates that chemotherapy alone significantly prolongs survival in PCL patients (3), while surgical intervention shows no prognostic benefit. Therefore, accurate diagnosis of PCL to avoid unnecessary thoracotomy carries paramount clinical significance in management.

## Data Availability

The raw data supporting the conclusions of this article will be made available by the authors, without undue reservation.
